# Notes and Notices

**Published:** 1894-05

**Authors:** 


### NOTES AND NOTICES.

How Shall We Dispose of the Homœopath?—The effect of a red flag on a bull is that of a lullaby compared with the fury of the "regular physician" when you flaunt the banner of Homœopathy at him.

If the homœopath had a first-rate system and lacked the intelligence to make it work, he would be more easily tolerated; but to adopt a laughable theory and then make a habit of deriving good results from it is extremely hard to forgive. We can understand the feelings of the old-school doctor who loses a patient in spite of all his efforts, when another and sicker patient across the street has the effrontery to recover under the foolish little pellets of the homœopath. But patients were ever unreasoning.

There are worldly spirits in every community who care more for their own recovery than for upholding the banner of the true faith. In fact, that willingness to die for a principle which characterizes the true hero seems lamentably on the wane. It must be speedily revived, however, if we are to keep the homœopath under water. Either he or the patient must be reformed.

There is a manifest willingness—we might say eager desire—among the medical profession to dispose of the homœopath by the gallows or the stake. But the times are hardly ripe for this happy disposition of the interloper. Yet it seems unwise to wait, as every year brings fresh recruits to the enemy's ranks. The problem will be forever solved when we can induce the patient to prefer an honorable death by an honorable system to an impertinent recovery by disrespectful methods. J. A. M.—*Life*.

International Journal of Surgery will remove on May 1st to the Downing Building, 106 and 108 Fulton Street, New York City.

A competitive examination for resident and associate-resident physicians of the Children's Homœopathic Hospital of Philadelphia will be held on *Thursday Evening, May 10th*, 1894, at eight o'clock, at the Hospital, 926 N. Broad Street. A large experience is afforded both resident physicians, in the out-patient department also, where about twelve thousand cases are prescribed for yearly at the medical and special clinics. Applications must be sent to Dr. Bushrod W. James, President of the Hospital, or to Dr. Jos. M. Reeves, President of the Medical Board, 926 N. Broad Street, Philadelphia, Pa.

The Homœopathic Medical Society of Ohio will hold its next annual meeting in Toledo, May 8th and 9th. Reduced railroad rates on all Ohio roads. An excellent scientific programme has been arranged, and the Toledo physicians will welcome visiting physicians, and provide for their enjoyment.

Membership blanks furnished in advance, on application to the Secretary,

Thos. M. Stewart, M. D.,
266 Elm Street, Cincinnati, O.

